# Does Differential Receptor Distribution Underlie Variable Responses to a Neuropeptide in the Lobster Cardiac System?

**DOI:** 10.3390/ijms22168703

**Published:** 2021-08-13

**Authors:** Audrey J. Muscato, Patrick Walsh, Sovannarath Pong, Alixander Pupo, Roni J. Gross, Andrew E. Christie, J. Joe Hull, Patsy S. Dickinson

**Affiliations:** 1 Department of Biology and Neuroscience Program, Bowdoin College, Brunswick, ME 04011, USA; ajmuscato98@gmail.com (A.J.M.); pwalsh88@pennmedicine.upenn.edu (P.W.); menghoutpong@gmail.com (S.P.); arpupow@gmail.com (A.P.); 2 USDA-ARS, Maricopa, AZ 85138, USA; roni.gross@usda.gov (R.J.G.); joe.hull@usda.gov (J.J.H.); 3 Pacific Biosciences Research Center, University of Hawaii at Manoa, Honolulu, HI 96822, USA; lobsterdoc@gmail.com

**Keywords:** allatostatin C, allatostatin C receptors, neuromodulation, cardiac ganglion, crustacean

## Abstract

Central pattern generators produce rhythmic behaviors independently of sensory input; however, their outputs can be modulated by neuropeptides, thereby allowing for functional flexibility. We investigated the effects of C-type allatostatins (AST-C) on the cardiac ganglion (CG), which is the central pattern generator that controls the heart of the American lobster, *Homarus americanus*, to identify the biological mechanism underlying the significant variability in individual responses to AST-C. We proposed that the presence of multiple receptors, and thus differential receptor distribution, was at least partly responsible for this observed variability. Using transcriptome mining and PCR-based cloning, we identified four AST-C receptors (ASTCRs) in the CG; we then characterized their cellular localization, binding potential, and functional activation. Only two of the four receptors, ASTCR1 and ASTCR2, were fully functional GPCRs that targeted to the cell surface and were activated by AST-C peptides in our insect cell expression system. All four, however, were amplified from CG cDNAs. Following the confirmation of ASTCR expression, we used physiological and bioinformatic techniques to correlate receptor expression with cardiac responses to AST-C across individuals. Expression of ASTCR1 in the CG showed a negative correlation with increasing contraction amplitude in response to AST-C perfusion through the lobster heart, suggesting that the differential expression of ASTCRs within the CG is partly responsible for the specific physiological response to AST-C exhibited by a given individual lobster.

## 1. Introduction

The neuronal networks that control rhythmic movements, such as locomotion and respiration, in both invertebrates and vertebrates [1,2,3] produce consistent, reliably patterned outputs. However, rhythmic behavior must often be altered in response to changing internal or external conditions; thus, flexibility is an innate characteristic of these pattern-generating networks. This flexibility in network outputs arises largely through the effects of neuromodulators (reviewed in [4,5,6,7,8], many of which are neuropeptides). Extensive modulation enables the same hard-wired network to produce a variety of rhythmic outputs; moreover, the effects of a given modulator can be state dependent (e.g., [9]) and differ both within and among individuals (e.g., [10]). Notably, cyclical changes in state, such as diurnal patterns, are an important and ubiquitous feature of the nervous system, although they are poorly understood. 

Among the mechanisms that have been demonstrated or proposed to account for the observed variability in neuronal response to modulators are (1) interactions with other modulators (e.g., [11,12]), (2) variation in the conductances of individual neurons within the neural network [13,14,15,16], and (3) the baseline level of activity within the network, i.e., “state dependence” (e.g., [9]). For example, several excitatory modulatory neurons, notably the anterior pyloric modulator (APM) neuron [17] and the modulatory proctolin neuron (MPN) [9], in the crustacean stomatogastric system have been shown to enhance the activity of the network to a lesser extent when the network is very active than when it is less active. Similarly, the effects of the C-type allatostatin peptides (AST-Cs) on networks of the stomatogastric system are more pronounced in slowly cycling preparations than in rapidly cycling ones; in this case, however, the peptide decreases activity, so that slowly cycling preparations become even slower and motor output is not altered in more active preparations [18]. In both instances, the action of the modulatory neuron can bias the system towards a specific output, but the extent of bias is state dependent.

In other cases, the mechanisms that underlie variable responses of neuronal networks are less clear. This is seen in the modulation of the neurogenic heartbeat of the American lobster, *Homarus americanus*, by the neuropeptide AST-C. The cardiac ganglion (CG), which is responsible for generating and controlling the heartbeat [19], is composed of only five motor neurons and four pre-motor neurons [19,20]. However, complex modulation by central nervous system inputs and neurohormones allows this simple cardiac system to remain responsive to the variety of physiological demands the lobster may face [3,6]. Similar to other decapod crustaceans, three AST-C peptide paralogs with neuromodulatory effects have been identified in *H. americanus* [21,22,23,24]: AST-C I (AST-C; pQIRYHQCYFNPISCF), AST-C II (AST-CCC; SYWKQCAFNAVSCFamide), and AST-C III (AST-CC; GNGDGRLYWRCYFNAVSCF; underscores indicate disulfide bonds). Although the AST-Cs can lead to increases in contraction amplitude and/or cycle frequency in some individuals [10,21], they more frequently elicit a decrease in both heart rate (network cycle frequency) and heart contraction amplitude. Individual responses to the AST-C peptides vary; the extent and direction of the changes elicited by AST-C result from the interactions of differential responses of the CPG in the cardiac ganglion [10] with the non-linear nature of the neuromuscular transform [25]. This suggests that some aspects of the CG differ in individuals that respond to AST-C with an increase in contraction amplitude and/or frequency compared to those that respond with a decrease. Because the effects of AST-C I and AST-C III are similar to one another, while also being more variable than those of AST-C II [21], we sought to determine the mechanisms that account for the variable responses of the two paralogs that elicit similar responses (i.e., AST-C I and AST-C III).

One possible source of the variable responses to AST-C peptides is a difference in the expression of receptors to these modulatory hormones [26,27]. AST-C receptor (ASTCR) diversity can vary depending on the species. While insects typically have a single receptor [24], multiple ASTCRs have been characterized in *Drosophila melanogaster* and *Aedes aegypti* [28,29]. In other lineages, ASTCR gene expansion is more varied—a lone receptor was reported in the Jonah crab, *Cancer borealis* [30], three in the sea star, *Asterias rubens* [31], and potentially four in the green shore crab, *Carcinus maenas* [32]. In *H. americanus*, previous analysis of a transcriptome produced from mixed tissues identified three putative ASTCRs, termed AST-CR I–III in that study [33] but renamed ASTCR1–3 here. ASTCR types, however, can exhibit varied spatiotemporal expressions [29,34]. Furthermore, given that sequence identity among proteins annotated as ASTCRs (also annotated as somatostatin receptor-like proteins and urotensin-2 receptor-like) can be limited (~ 30%), the pool of potential hits identified via BLAST analyses when similar sequences are used as queries is likewise limited. Consequently, to reassess the ASTCR complement in *H. americanus*, we expanded the ASTCR query dataset to mine multiple tissue-specific transcriptomes, including the eyestalk ganglia [35], brain [36], and CG [37]. This approach led to the identification of a fourth putative ASTCR (ASTCR4). 

The presence of multiple receptor-like sequences suggests the possibility that each CG may express any combination of the four predicted ASTCRs. One combination of receptors might lead to variation in receptor hetero/homodimerization with subsequent modulatory effects that decrease heart contraction amplitudes, whereas a different combination of receptor oligomers could lead to an increase. For this to be the case, however, the predicted ASTCRs need to be functionally expressed in the CG. To determine if that was the case, we combined transcriptome data mining with tissue-specific PCR-based cloning to confirm the presence of all four predicted ASTCR transcripts in the CG. To provide support for the initial BLAST-based annotations, predicted receptor sequences were compared with previously characterized ASTCRs as well as with sequences for receptors of somatostatin, the mammalian homolog of AST-C [28,29,38,39,40]. We then used a heterologous insect cell expression system to examine cellular localization, binding potential, and functional activation. ASTCR1 and ASTCR2 each showed the characteristics of a G protein-coupled receptor (GPCR). Similar to the data reported for the honeybee (*Apis mellifera*) ASTCR [39], intracellular cAMP levels in cells stably expressing each receptor decreased in response to AST-C I, consistent with a G_αi/o_ signaling pathway. In contrast, neither ASTCR3 nor ASTCR4 localized to the plasma membrane. 

To determine if differential distribution of the ASTCRs might contribute to the varied responses of the cardiac systems to AST-C paralogs, we used high-throughput RNA sequencing to assess the CG transcript profile in hearts whose physiological responses to AST-C had been recorded. Intriguingly, the expression levels of ASTCR1, but not those of the other three receptors, showed a significant negative correlation with the amplitude of the cardiac response to AST-C I, suggesting that the response might be in part determined by the distribution of this receptor type.

## 2. Results

### 2.1. Transcripts Encoding Four Putative ASTCR Homologs Are Predicted in H. americanus Transcriptomic Datasets

**Figure 1:**
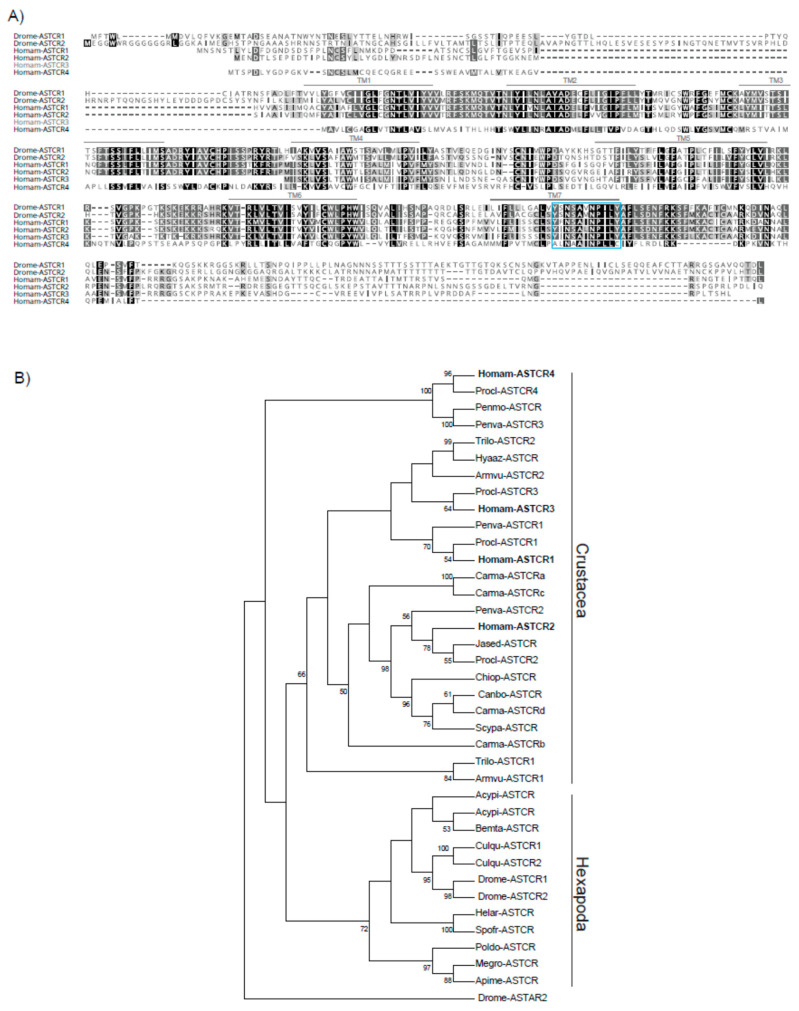
Sequence and phylogenetic analyses suggest ASTCR4 belongs to a more evolutionarily distant group of conserved receptors. (**A**) MUSCLE-based multiple sequence alignment of *H. americanus* ASTCRs and functionally characterized *Drosophila melanogaster* AST-C receptors (AAF49259.2 and AAN11677.2). The locations of predicted transmembrane (TM) domains, indicated by a gray overline, are based on the *D. melanogaster* ASTCR1 sequence. The TM7 motif characteristic of somatostatin/AST-C-type receptors is indicted by a blue box. (**B**) Maximum likelihood tree of ASTCR-like proteins from diverse arthropod species. *H. americanus* ASTCRs are indicated in bold. The bootstrap consensus tree was inferred from 1000 replicates; shown next to the branches is the percentage of replicate trees in which the associated taxa clustered. Branches corresponding to partitions reproduced in less than 50% of bootstrap replicates have been collapsed. Species abbreviations and accession numbers are listed in Table S6.

A more robust data mining approach than the one previously employed, which utilized a single transcriptome, was used to re-evaluate *H. americanus* ASTCR diversity. The inclusion of multiple datasets did not significantly expand the complement of ASTCRs as the revised search returned only a single new sequence (Homam-ASTCR4) in addition to the three receptor sequences reported previously. Conceptual translation of all four transcripts revealed sequence motif signatures consistent with GPCRs, including multiple transmembrane (TM) domains, an extracellular N terminus, an intracellular C terminus, and potential *N*-glycosylation sites in the N-terminal loop (Table S1). Based on alignments, the three receptors identified previously exhibited 57–66% sequence identity, whereas Homam-ASTCR4 conservation was more limited at ~20% (Figure 1A). BLASTx analyses of the putative Homam-ASTCRs revealed significant similarities with sequences annotated as somatostatin and/or AST receptor-like, including Homam-ASTCR4, which had weaker, but still highly significant *E* values (Table S2). To assess the relatedness of the Homam-ASTCRs to other somatostatin and/or AST receptor-like annotated sequences, we performed a maximum likelihood-based phylogenetic analysis using 34 sequences from 21 arthropod species, including representatives of the Crustacea and Hexapoda. The resulting consensus tree placed the Homam-ASTCRs in receptor-specific crustacean clades, indicating that any gene duplication event occurred in an evolutionary ancestor common to both crustaceans and hexapods (Figure 1B). Homam-ASTCR4 unexpectedly sorted to a potentially more ancient clade that segregated away from the other ASTCRs and included sequences annotated as urotensin-2 receptor-like and a *Procambarus clarkii* ASTCR. Trees with similar topologies that likewise suggested evolutionary divergence of the Homam-ASTCR4 clade were generated using the neighbor joining and minimum evolution methods (Figure S1A,B). Given this unexpected location in the phylogenetic tree, we further examined the relationship of the Homam-ASTCRs in relation to the full complement of peptide receptors identified in *D. melanogaster* [41,42] and *C. borealis* [30]. ASTCRs from the three species clustered in their own receptor-specific clade (Figure S2), further supporting annotation of the Homam-ASTCR4 sequence. Additionally, the 11-amino acid somatostatin/AST-C-type receptor signature motif (YANSCANPI/VLY) reported in TM7 [29,43] is clearly present in all of the Homam-ASTCRs (Figure 1A). Non-conservative substitutions (Ala for Tyr1 and Cys for Tyr11) of the terminal Tyr residues, however, are present in the Homam-ASTCR4 motif and appear to be characteristic in the sequences that comprise the Homam-ASTCR4 clade (Figure 1B).

Consistent with the initial identification of Homam-ASTCR3 [33], no transcripts were identified among the datasets searched that would extend the presumptive partial sequence (i.e., absence of clear start site). Attempts to identify a viable alternative start codon via 5′RACE methods (Hull, unpublished data) were unsuccessful. Furthermore, BLASTp analyses of the predicted sequence based on the first in-frame Met residue yielded a genome-derived hit (MPC89394.1) from the gazami crab (*Portunus trituberculatus*) with 84% sequence similarity and an identical predicted start site. The encoded proteins are smaller than typical GPCRs, with fewer predicted TM domains and/or uncharacteristically shorter domain segments and limited predictive support for localization at the plasma membrane (Table S1).

To assess potential ASTCR transcript abundance in the CG, the longest identified Homam-ASTCR transcriptomic sequence was used to query a CG-specific dataset (PRJNA412549). BLASTn results identified transcripts with 100% identity to full-length Homam-ASTCR2 and Homam-ASTCR4 (Table S3). Queries using the Homam-ASTCR1 and 3 sequences returned hits with 82% and 76% identity, respectively, with the Homam-ASTCR2 transcript. The absence of transcripts for Homam-ASTCR1 and 3 could indicate that neither is expressed in the CG or, alternatively, that the transcripts may be conditionally expressed.

### 2.2. Transcripts Encoding the Four Putative ASTCRs Are Expressed in the CG

**Figure 2:**
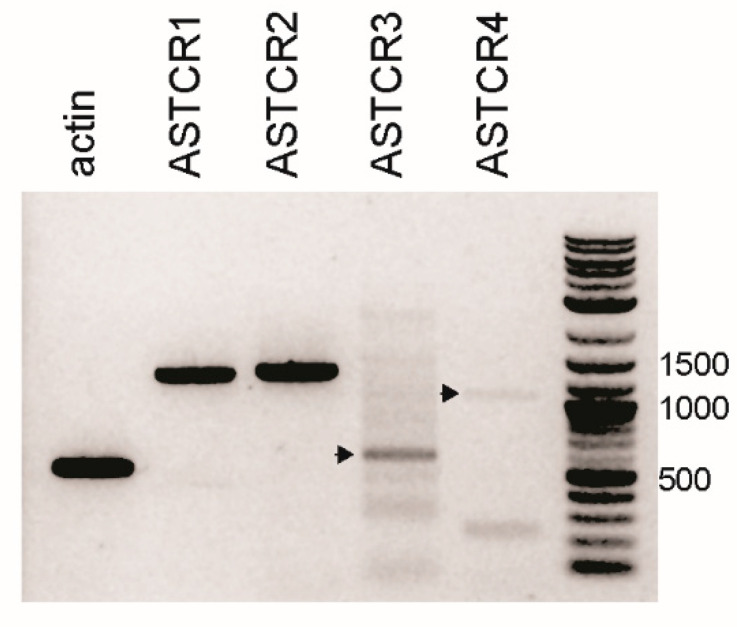
Transcripts encoding ASTCRs are expressed in the CG. RT-PCR amplification of full-length open reading frames encoding the four respective *H. americanus* ASTCRs from pooled CG cDNAs. Expected amplimer sizes: ASTCR1—1263-bp; ASTCR2—1305-bp; ASTCR3—753-bp (arrowhead); and ASTCR4—1032-bp (arrowhead).

Using primers designed to amplify the full-length open reading frames, all four Homam-ASTCRs were amplified from pooled CG cDNAs (Figure 2), suggesting that the absence of the Homam-ASTCR1 and 3 sequences in the CG-specific transcriptome reflected limited depth of the dataset. Primers for ASTCR3 yielded multiple non-specific products in addition to a strong band of the predicted size (753-bp). ASTCR4 primers generated two relatively faint amplimers, one of the predicted size (1032-bp) and a much smaller non-specific product. Although the ASTCR transcripts are clearly expressed in the CG, the amplification across multiple biological replicates was inconsistent (data not shown), suggesting that the receptors may be conditionally expressed, as also suggested by the limited representation in the CG-specific transcriptomic dataset.

### 2.3. ASTCR1 and ASTCR2 Localize at the Cell Surface and Specifically Bind AST-C Peptides

**Figure 3:**
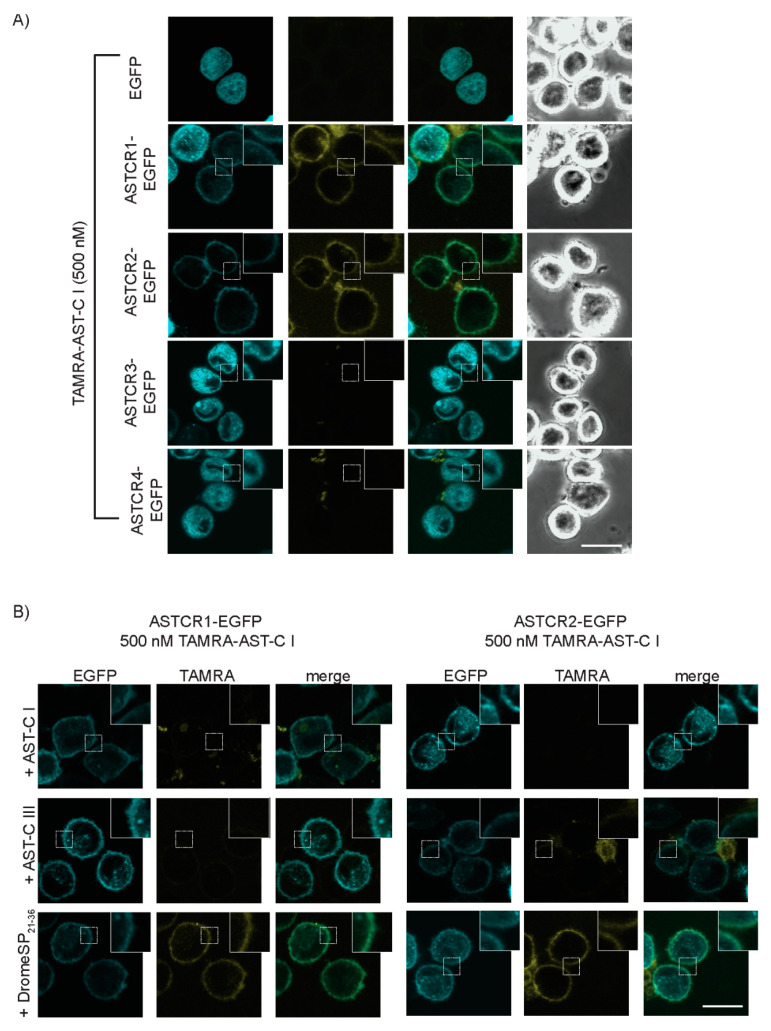
Fluorescent chimeras of select ASTCRs localize at the cell surface and specifically bind AST-C peptides. (**A**) Live cell imaging of Tni insect cells transiently expressing fluorescent EGFP chimeras of the respective *H. americanus* ASTCRs. Cells were incubated (1 h at 4 °C) with a fluorescent analog of *H. americanus* AST-C I labeled at the N terminus with carboxytetramethylrhodamine (TAMRA). (**B**) Live cell imaging competitive binding assay of Tni cells transiently expressing fluorescent EGFP chimeras of *H. americanus* ASTCR1 or 2. Cells were incubated as before, but also in the presence of 5 µM unlabeled *H. americanus* AST-C I, AST-C III, or a *D. melanogaster* sex peptide fragment corresponding to residues 21–36 (DromeSP21–36). Insets: magnification of plasma membrane fluorescence. All cells were imaged in the presence of 0.45 M sucrose (inhibition of clathrin-mediated receptor internalization) using filter sets for EGFP (Em = 489 nm/Ex = 510 nm) and TRITC (Em = 546 nm/Ex = 579 nm) with EGFP and TAMRA fluorescence pseudo-colored cyan and yellow, respectively. Co-localization of the two signals, depicted in green in the merged images, supports receptor-dependent binding. Images are representative of cells encompassing multiple independent transfections. Scale bar = 20 µm.

As transducers of extracellular signals, GPCRs typically localize to the lipid bilayer that comprises the cell membrane. Consistent with this role, Homam-ASTCR1, 2, and 4 are predicted with high confidence to localize at the plasma membrane (Table S1). In contrast, the predicted localization of Homam-ASTCR3 was largely indeterminate. To empirically test these predictions, fluorescent chimeras of the ASTCRs, which had enhanced green fluorescent protein (EGFP) fused to the respective C terminal tails, were transiently expressed in cultured Tni insect cells (ovarian cells derived from the cabbage looper, *Trichoplusia ni* [44]); their cellular localization was examined via confocal microscopy (Figure 3A). Cells expressing EGFP alone exhibited a uniform fluorescence signal that was dispersed throughout the cytoplasm. In contrast, the ASTCR1-EGFP and ASTCR2-EGFP signals were, as predicted, predominantly localized at the cell surface. Conversely, the ASTCR4-EGFP chimera failed to localize at the cell surface despite a prediction of plasma membrane localization. ASTCR3-EGFP likewise showed no evidence of cell surface localization. Instead, the fluorescent signal for both constructs was more reminiscent of ER localization [45], suggesting that neither is a functional GPCR in the traditional sense. An EGFP chimera of the *P. clarkii* ASTCR4 homolog, which sorted to the ASTCR4-specific clade (Figure 1B), also exhibited diffuse intracellular fluorescence with no evidence of cell surface localization (Figure S3), suggesting that this receptor group may be intracellularly retained when expressed in insect cells. Impaired receptor trafficking due to the chimeric nature of the expressed receptor, however, cannot be ruled out. 

To assess the ligand binding potential of the ASTCR-EGFP chimeras, confocal microscopy was used to examine localization of a synthetic AST-C I analog labeled at its N terminus with the red fluorescent dye carboxytetramethylrhodamine (TAMRA). No evidence of ligand binding was observed in cells expressing EGFP alone (Figure 3A), indicating the absence of an endogenous receptor capable of binding the tested ligand. Similarly, TAMRA-derived signals were absent in cells expressing either ASTCR3 or ASTCR4. While the lack of observable binding is consistent with observations that neither receptor translocates to the cell surface, it does not preclude the possibility that the AST-C I analog is not recognized by the two receptors. In contrast, cells expressing ASTCR1-EGFP and ASTCR2-EGFP displayed a clear TAMRA signal that localized at the cell surface, and merged images of the two fluorescence channels showed clear co-localization of the respective signals consistent with the receptor–ligand binding (Figure 3A). Furthermore, the TAMRA-ASTC-C I signal was largely abolished when incubated with excess unlabeled *H. americanus* AST-C I or AST-C III (Figure 3B). This effect was not observed in cells incubated with an analog of an unrelated insect peptide, *D. melanogaster* sex peptide (an AST-B receptor ligand). These results demonstrate that the observed localization of TAMRA-ASTC-C I is the result of a specific, reversible interaction with ASTCR1 and ASTCR2, thereby confirming both as bona fide AST-C receptors.

### 2.4. ASTCR Localization and Ligand Binding Are Not Affected by the Addition of EGFP 

**Figure 4:**
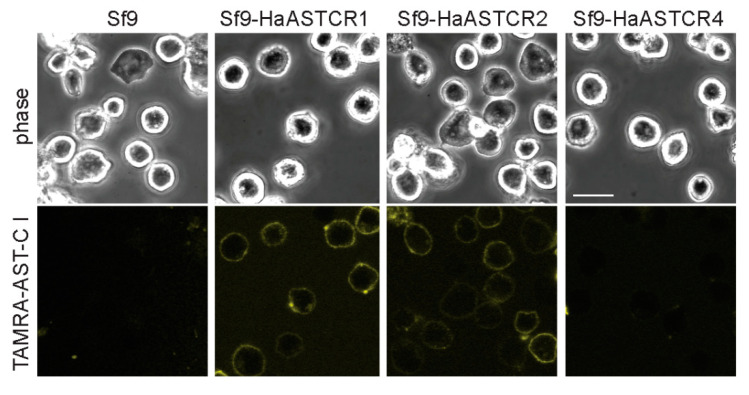
ASTCR localization and binding is not EGFP dependent. Live cell imaging of Sf9 insect cells alone or stably expressing *H. americanus* ASTCR1, 2, or 4. Cells were incubated (1 h at 4 °C) with the TAMRA-AST-C I analog and imaged in the presence of 0.45 M sucrose as before using a TRITC filter (Em = 546 nm/Ex = 579 nm) with TAMRA fluorescence pseudo-colored yellow. Scale bar = 20 µm.

Given the unexpected lack of membrane localization of the Homam-ASTCR4 chimera, we sought to repeat the heterologous cell expression study using polyclonal Sf9 insect cells (a cell line derived from the ovaries of the fall armyworm, *Spodoptera frugiperda* [46]) stably expressing non-tagged versions of Homam-ASTCR1, 2, and 4. After confirming expression of the transgene in the respective polyclonal lines (Figure S4), wildtype, non-transfected Sf9 cells and polyclonal cells were incubated with TAMRA-labeled AST-C I, and localization of the fluorescent signals was assessed using confocal microscopy. No TAMRA-derived fluorescent signal was observed in the wildtype Sf9 cells or polyclonal cells expressing Homam-ASTCR4 (Figure 4), suggesting that the lack of cell surface trafficking observed for ASTCR4 was not an artifact of fusion with the EGFP sequence. In contrast, clear fluorescent signals were evident at the surface of cells stably expressing Homam-ASTCR1 or Homam-ASTCR2 (Figure 4).

### 2.5. Stably Expressed Homam-ASTCR1 and 2 Are Activated by AST-C I and AST-C III

**Figure 5:**
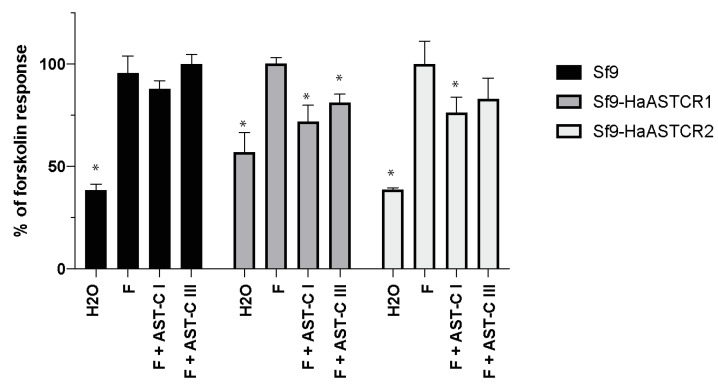
ASTCR1 and ASTCR2 are activated by AST-C peptides. Intracellular cAMP levels in Sf9 insect cells alone or stably expressing *H. americanus* ASTCR1 or 2 were measured via a cAMP ELISA kit following incubation with H_2_O alone, 10 µM forskolin (F; adenylate cyclase activator), or forskolin + synthetic *H. americanus* AST-C I or AST-C III. Values have been normalized relative to the maximal forskolin response for each cell set. Bars represent mean +/- SEM values of cells assayed in triplicate. Asterisks (*) indicate values that are significantly different from the forskolin-activated response (ANOVA, with uncorrected Fisher’s LSD, *p* < 0.05; *n* = 4).

ASTCRs have been reported to couple through G_αi/o_ subunits to trigger a reduction in intracellular cAMP levels [28,39]. To examine this aspect of AST-C activation, the polyclonal Sf9 cells stably expressing Homam-ASTCR1 or Homam-ASTCR2 were treated with forskolin, an adenylate cyclase activator that induces cAMP, or a mixture of forskolin and Homam-AST-C I or III (Figure 5). To facilitate cross-experiment comparisons, measurements were normalized and presented as a percentage of the maximal forskolin response. As expected, forskolin stimulation significantly increased cAMP levels relative to H_2_O alone in all cells assayed. For wildtype Sf9 cells, the addition of either *H. americanus* AST-C peptide in conjunction with forskolin resulted in a cAMP response that was indistinguishable from forskolin alone, suggesting the absence of an endogenous ASTCR. In cells stably expressing either of the two ASTCRs, however, a statistically significant reduction in the cAMP response was observed when cells were incubated in the presence of AST-C I. A similar reduction was observed in cells expressing Homam-ASTCR1 in response to AST-C III, whereas no reduction in response to AST-C III was observed in cells expressing Homam-ASTCR2. Taken together, the data indicate that these two Homam-ASTCRs are functional receptors activated by AST-C peptides.

### 2.6. Differential Expression of ASTCR1 Is Correlated with Physiological Response

**Figure 6:**
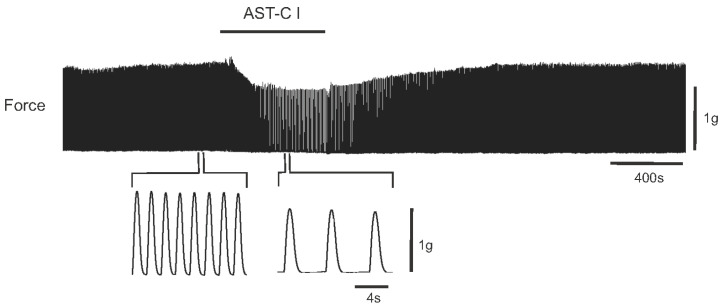
AST-C I modulates the contraction amplitude of the *H. americanus* heartbeat. In this sample recording, the perfusion of AST-C I through the heart caused the contraction amplitude to decrease; however, AST-C I can also cause an increase in contraction amplitude. The heartbeat returned to the baseline amplitude when the peptide was washed out.

After identifying and characterizing the four ASTCRs, we sought to characterize their expression levels in the CG across individuals in order to determine whether there was a correlation between physiological activity and the expression of putative AST-C receptors. Therefore, we recorded the responses of lobster hearts to AST-C I and III, then isolated CG RNA from the same lobsters, and assessed transcript abundance in each CG using RNA-seq (Figure 6).

**Figure 7:**
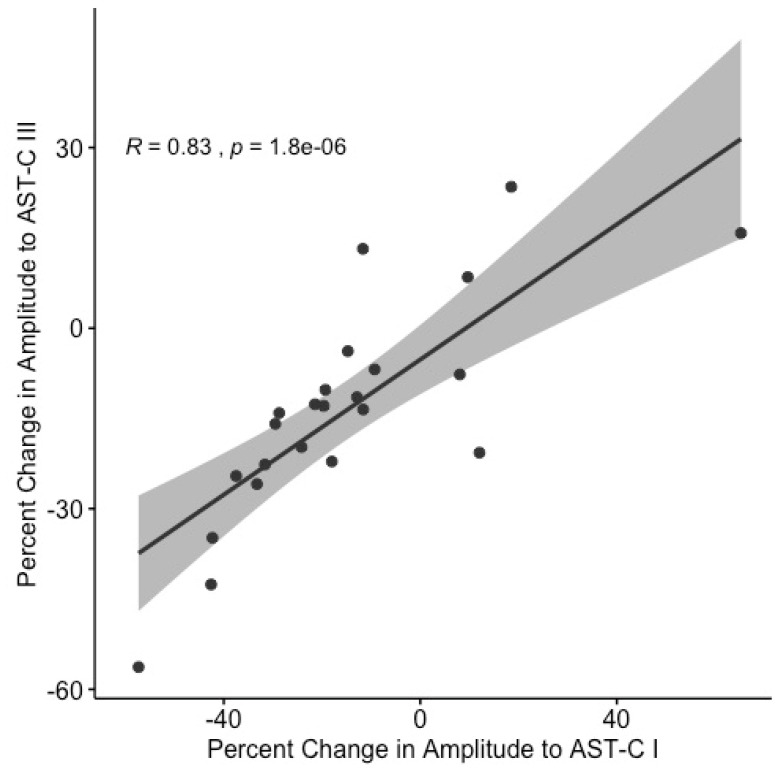
Physiological responses of individual lobsters to AST-C I and AST-C III are strongly positively correlated (Spearman correlation, R = 0.83, *p* < 0.0001). Physiological responses were measured as percent change in contraction amplitude in response to perfusion of the peptide. RNA for sequencing was extracted from the CGs of the lobsters used in this analysis. One outlier was omitted using the GraphPad Prism ROUT method (*n* = 23).

To test the hypothesis that differential receptor distribution underlies the varied responses of individual hearts to AST-C peptides, we analyzed the RNA-seq data for correlations between the expression of the four receptors and the physiological response of each lobster. First, we confirmed that there was a strong positive correlation between the physiological responses of individual lobsters to AST-C I and to AST-C III (Figure 7), as had been seen in previous studies [21]. We inferred that the factors driving the response to one AST-C isoform could also drive the physiological response to the other isoform. Given this correlation, bioinformatic analyses of the RNA-seq data were performed using the response to AST-C I as indicative of the response to both peptides.

**Figure 8:**
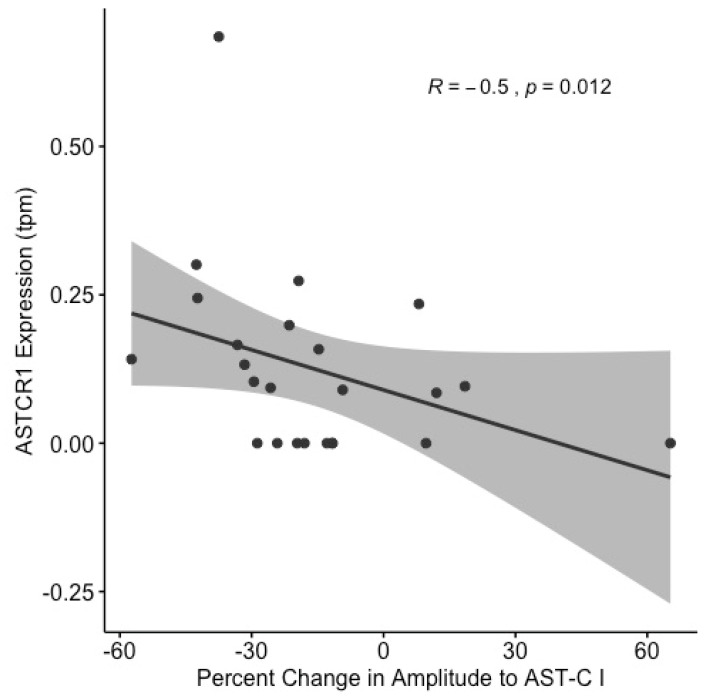
Differential expression of ASTCR1 is partly responsible for the great individual variability in physiological responses to AST-C. The percent change in amplitude to AST-C I and the CG expression (transcripts per million) of ASTCR1 are significantly negatively correlated (Spearman correlation, R = −0.5; *p* = 0.012; *n* = 23 lobsters).

The expression of ASTCR1 was negatively correlated with the change in contraction amplitude elicited by AST-C I perfusion through the heart (Spearman correlation; R = –0.5, *p* = 0.012; Figure 8). However, the expression levels of the other three putative receptors were not significantly correlated with the physiological response (Table S4). These data suggest that differential expression of ASTCR1 is a factor in determining the change in contraction amplitude elicited by perfusion with AST-C I, but that the expression levels of the other receptors are not involved. This finding, however, did not preclude the possibility that the differences in response among individuals might be determined in part by the relative expression of the receptors to one another, rather than by the expression level of individual receptors. To determine whether this was the case, Spearman correlations of all six pairs of receptor expression ratios (i.e., ASTCR1:ASTCR2, ASTCR1:ASTCR3, etc.) to the AST-C I physiological response were performed. The ratio of expression of ASTCR1 to ASTCR4 showed a trend towards a correlation (p = 0.075) with the physiological response to AST-C I (Figure S5). This suggests the possibility that ASTCR4, which appears, at least in our heterologous expression system, to be an atypical non-plasma membrane localized GPCR, might be involved in the mechanism determining the directionality and amplitude of the response alongside ASTCR1. None of the other correlations performed between physiological response and all other possible receptor expression ratio combinations neared significance.

## 3. Discussion

Using in silico analysis of the *H. americanus* transcriptomes generated from brain tissue, eyestalk ganglia, and CG, we identified four putative ASTCR-like sequences. We hypothesized that a combinatorial expression pattern of these putative receptors might underlie the range of physiological responses elicited by perfusion of different AST-C peptide paralogs. We thus sought to confirm the expression and identity of the putative receptor-like sequences, characterize the predicted amino acid structures, and assess functionality. Finally, we sought to identify the mechanism by which AST-C I and AST-C III potentially elicit a wide range of individual responses in contraction amplitude of the lobster heartbeat.

Full-length open reading frames of the four putative ASTCRs were amplified from pooled CG cDNAs; all of the identified sequences were nearly identical to the transcriptomic sequences. Although ASTCR1 and ASTCR2 transcripts were more readily amplified in the pooled CG sample compared to ASTCR3 and ASTCR4, this may simply reflect differences in primer efficiency rather than differential transcript expression within the CG. Quantitative analyses using individually isolated CGs (or even motor and pre-motor neurons) would need to be performed to confirm this. Regardless, the expression of all four transcripts within the CG is consistent with the hypothesis that differential or combinatorial expression of these receptor transcripts may explain observed differences in physiological response.

Although ASTCRs 1–3 were identified in an earlier transcriptomic mining effort [33], their characterization was largely limited to sequence alignments; moreover, the ASTCR3 transcript was predicted to be incomplete. The more extensive analyses reported here support the initial structure-based annotations and suggest that the original ASTCR3 may encode a full-length receptor with features typical of 7TM domain GPCRs, albeit with smaller loop regions. Consistent with predictions, heterologously expressed ASTCR 1 and 2 localized at the plasma membrane and coupled through a G_αi/o_ signaling pathway when activated by AST-C I and to a lesser extent AST-C III. Similar effects on the cAMP secondary messenger system were reported for the honeybee ASTCR [39] and fruit fly (*Drosophila melanogaster*) ASTCRs (Drostar 1 and 2), which presumably activate GIRK currents via G_αi/o_ subunits [28]. Vertebrate somatostatin receptors, recognized as homologs of the arthropod ASTCRs [28,40,47], similarly couple through the G_αi/o_ pathway [48]. Activation of both Homam-ASTCRs by the same ligand (i.e., AST-C I and to a lesser extent AST-C III) is not with precedent as both of the ASTCRs identified in mosquitos and the fruit fly were likewise activated by AST-C I (also referred to as AST-C/PISCF allatostatin), with little difference in potency between the respective receptors in each species [28,29]. Similarly, the activation of a single ASTCR by two AST-C peptides was reported for the honey bee ASTCR [39] and the red flour beetle (*Tribolium castaneum*) ASTCR [49], suggesting that the lack of receptor discrimination pre-dates the evolutionary split between Crustacea and Hexapoda. Different spatiotemporal expression of the respective ASTCRs could account for the similar activation profiles and may indicate functional roles that mediate distinct AST-C biological effects. In contrast to Homam-ASTCR 1 and 2, ASTCR3 did not localize to the cell surface (nor was it predicted to do so). Although the poor trafficking may be an artifact of the chimeric construct, the encoded receptor may indeed be truncated as suggested previously [33]. Additional targeted sequencing will be needed to fully resolve this issue.

Unlike the other ASTCRs, Homam-ASTCR4 exhibits greater structural and phylogenetic divergence. It, however, still aligns with sequences annotated as ASTCRs when compared with species-specific complements of arthropod peptide receptors. This variance might reflect the common ancestral origin proposed for the vertebrate somatostatin and urotensin II receptors, both of which bind small disulfide-bridged peptides with shared structural features [48]. In support of this, the arthropod Homam-ASTCR4 showed highest similarity with arthropod receptors annotated as urotensin II receptor-like proteins. Despite a prediction of plasma membrane localization, the fluorescent Homam-ASTCR4 chimera did not localize at the cell surface; moreover, no evidence of ligand binding was observed in cells stably expressing an untagged version of the receptor. This could indicate different ligand specificity for the receptor relative to Homam-ASTCR 1 and 2. Indeed, urotensin II receptors from the western clawed frog were reportedly not activated by somatostatin [50]; however, this ligand discrimination does not always appear to be the case, as various somatostatin receptor subtypes can be activated by urotensin II [51]. Alternatively, cell surface trafficking of Homam-ASTCR4 may require the presence of regulatory proteins (e.g., ER chaperones, accessory proteins, and/or receptor activity modifying proteins) present in cells that comprise the CG, but absent in our insect cell system [52,53].

After characterizing and confirming the function of the putative ASTCRs in the lobster CG, we sought to answer the biological question at hand: what is the mechanism underlying the variability of individual responses to AST-C peptides? Previous work has identified this AST-C response variability and ruled out the possibility of state dependence, as measured by baseline activity, as a biological mechanism [10,21]. Through a combination of physiological and bioinformatic techniques, we identified differential expression of ASTCR1 across lobster CGs as a factor involved in determining the response of heartbeat amplitude to AST-C I and III. Notably, levels of ASTCR1 expression in the lobster CG were correlated with the individual physiological response to AST-C I perfusion. In contrast, the expression levels of the other putative ASTCRs (Homam-ASTCR2-4) were not significantly correlated with physiological response. The presence of differential receptor expression in a neural network is consistent with other findings in crustacean nervous systems; for example, the crustacean cardioactive peptide (CCAP) receptor is differentially expressed across neuronal types in the stomatogastric ganglion (STG) of the crab *Cancer borealis*, as well as among individuals [54].

Although significant AST-C response correlations were limited to the expression level of ASTCR1, we cannot rule out the possibility that the other receptors play a role in the response to AST-C, or that there are interactions between the different AST-C receptors that were not reflected in significant correlations between physiological responses and ASTCR ratios. Interestingly, mammalian somatostatin receptors (SST2 and SST5) have been shown to modulate each other, suggesting that this possibility also exists for ASTCR interactions [55]. Numerous studies have also implicated receptor dimerization in the translocation of some GPCRs through the secretory pathway [53,56,57,58]. As such, it could be that ASTCR4 cell surface localization requires heterodimerization with one of the other ASTCRs. If this were the case, interactions between the receptors could be important in determining the physiological response of the CG, as receptor dimerization has been shown to modulate receptor activity [59,60,61]. Alternatively, ASTCR4 may indeed be poorly trafficked in vivo and exert physiological effects on AST-C function via endosomal compartments [62] or by impacting ASTCR1 expression at the cell surface. Regardless, it is becoming increasingly clear that combinatorial expression of receptors in a particular cell type or tissue can collectively influence physiological responses via differential signaling [63]. Indeed, the importance of multiple receptors responding to a neuropeptide and being individually responsible for a component of the response can be seen in the effects of serotonin on the pyloric network of the spiny lobster [64].

One limitation of the RNA sequencing and bioinformatics workflow in this study was the pooling of all nine CG neurons into a single sample, instead of separating the different cell types. It is possible that the differential expression between the motor neurons and pre-motor neurons among individual lobsters was masked due to the experimental design. Previous literature has shown, for example, that receptors for the neuropeptide myosuppressin (MSR) are differentially expressed between cell types, with MSR-II and MSR-III having higher expression in the motor neurons and MSR-IV having higher expression in the pacemaker neurons [65]. Thus, there is the possibility that not only are the expression levels of the ASTCRs in the CG important in determining an individual lobster’s response to AST-C, but also that differences in where those receptors are expressed among lobsters could contribute to this wide variation in response.

Further research also needs to be conducted to investigate the potential cause of the individual variation that is observed in response to perfusion of AST-C I and III. One possibility is that the molt stage of the lobster dictates the direction of the response in contraction amplitude of the heartbeat. This would lead us to hypothesize that the differential expression of ASTCR1 in the CG is dependent on the molt stage and thus serves as a mechanism that enables the lobster to respond differently to the same peptide across various physiological conditions. Current crustacean literature is consistent with this hypothesis, as a number of receptors have been found to be differentially expressed across the molt stage in different systems, such as the Y-organs and the STG [32,54]. Further work investigating possible connections between ASTCR expression in the CG and the molt cycle might help elucidate the physiological significance of AST-C and further our understanding of the physiological importance of the individual variation between lobsters.

**Figure 9:**
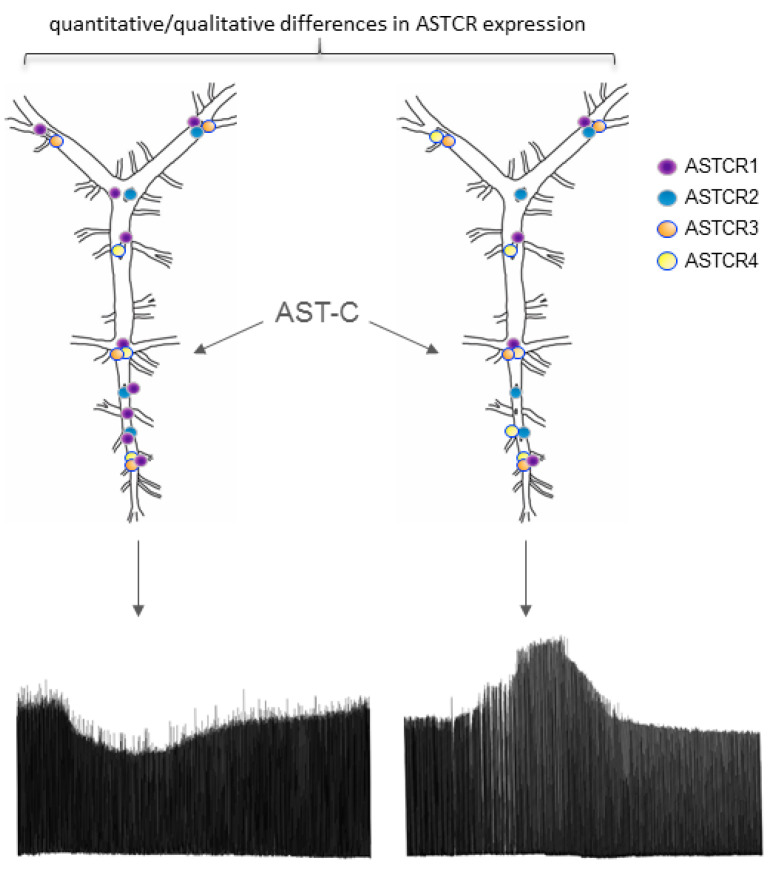
Differential receptor distribution partially explains individual variation in response to AST-C. This schematic shows two different cardiac ganglia (CG) with differential receptor expression; importantly, the CG on the left has a higher expression of ASTCR1 than the CG on the right. As explained by the correlation with physiological response, the CG with higher ASTCR1 expression responds to AST-C with a decrease in contraction amplitude, while the CG with lower ASTCR1 expression responds to AST-C with an increase in contraction amplitude. Notably, there is also a nonsignificant correlation with the ratio of ASTCR1:ASTCR4 expression and physiological response, while ASTCR2 and ASTCR3 expressions remain constant. ASTCR3 and ASTCR4 are depicted with blue lines to indicate potential intracellular, non-ligand binding functionalities.

As presented in the current paper, the identification and functional characterization of the four ASTCRs in the *H. americanus* CG provides insights into the mechanism by which the CG responds to AST-C. This allows us to ask biological questions such as how the observed variation in response to the peptide among lobsters could be explained. The differential expression of ASTCR1 across individual lobsters with responses to AST-C along a continuum elucidates how that variation might arise mechanistically. Thus, we present a mechanism, illustrated schematically in Figure 9, by which a pattern-generating network can respond to the same peptide modulator in opposing ways, depending on individual receptor expression.

## 4. Materials and Methods

### 4.1. Lobsters

Wild-caught American lobsters (*Homarus americanus*) were purchased in Brunswick, Maine and housed at Bowdoin College in tanks of recirculating seawater kept at 10–12 °C. The lobsters were kept on a 12-hour light, 12-hour dark cycle and were fed chopped shrimp and squid weekly. Both male and female soft- and hard-shell lobsters (approximately 500 g, corresponding to an age of approximately 5–10 years) were used for the experiments.

### 4.2. Transcriptome Data Mining and Associated Transcript Analyses

To reassess the complement of ASTCRs in *H. americanus*, the three Homam-ASTCRs previously identified and sequences from six additional arthropods annotated as ASTCRs or somatostatin receptor-like proteins (Table S5) were used with the online program tBLASTn (National Center for Biotechnology Information, Bethesda, MD; http://blast.ncbi.nlm.nih.gov/Blast.cgi, accessed on 7 June 2021) to query *H. americanus* specific transcriptome shotgun assembly (TSA) datasets (Bioproject accessions 412549, 379629, 338672, and 300643). The hits were then re-evaluated via BLASTx against the nr database to confirm initial ASTCR or ASTCR-like annotations. This approach identified the three ASCTRs reported previously as AST-CR I, AST-CR II, and AST-CR III [57], which we now refer to as ASTCR1-3, as well as a fourth putative receptor, ASTCR4. The nucleotide sequences of the respective ASTCRs were then searched against a CG-specific dataset [37]. The longest transcripts were conceptually translated and scanned for defined protein motifs using ScanProsite [66] and the HMMscan module on the HMMER webserver [67]. In addition, the respective sequences were examined for the signature sequence in the seventh transmembrane domain (YANSCANPI/VLY) that is characteristic of somatostatin receptors and ASTCRs [29]. Transmembrane domains were predicted using TOPCONS [68]. *N*-glycosylation sites were predicted for each full-length receptor using the NetNGlyc 1.0 server (https://services.healthtech.dtu.dk/service.php?NetNGlyc-1.0, accessed on 7 June 2021), and common motifs were annotated if identified. Cellular localization predictions were made with WoLF pSORT [69]. All receptor alignments and percent identities were determined using MUSCLE [70] with default settings in Geneious Prime 2020.1.2 [71]. 

To examine the phylogenetic relatedness of the predicted Homam-ASTCRs with ASTCR and ASTCR-like sequences from diverse arthropods species (Table S6), a multiple sequence alignment was generated via MUSCLE using default settings implemented in Geneious Prime 2020.1.2 [71]. The evolutionary history was inferred via the maximum likelihood method with the Le and Gascuel 2008 model [72] in MEGA X [73]. Initial tree(s) for the heuristic search were obtained automatically by applying Neighbor-Join and BioNJ algorithms to a matrix of pairwise distances estimated using the JTT model, and then selecting the topology with superior log likelihood value. A discrete gamma distribution was used to model evolutionary rate differences among sites (5 categories (+G, parameter = 0.9448)). The analysis involved 38 amino acid sequences. All positions with less than 95% site coverage were eliminated, and fewer than 5% alignment gaps, missing data, and ambiguous bases were allowed at any position (partial deletion option). This resulted in a total of 295 positions in the final dataset. Neighbor joining [74] and minimum evolution [75] approaches yielded trees with similar topologies (Figure S1A,B).

### 4.3. PCR-Based Amplification and Cloning of Transcripts for H. americanus ASTCR1-4 from Pooled CG cDNAs

To confirm the presence of ASTCR transcripts in the CG, total RNAs were purified from four biological replicates of the *H. americanus* CG (ten ganglia per replicate) as described previously [37]. cDNAs were generated from ~100 ng of DNase I-treated total RNAs using Superscript III Reverse Transcriptase (ThermoFisher Scientific, Waltham, MA, USA) and custom made random pentadecamers (IDT, San Diego, CA, USA). Full-length open reading frames of the four predicted receptors were amplified using Sapphire Amp Fast PCR Master Mix (Takara Bio USA Inc., Mountain View, CA, USA) in 20 μL reaction volumes containing 1 μL cDNA and primers (Table S7) designed to the respective start and stop codons. As a positive control, a 504-bp fragment of *H. americanus* actin (FJ217215) corresponding to nt 439-942 was likewise amplified. Thermocycler conditions consisted of 95 °C for 2 min followed by 37 cycles at 95 °C for 0:20 min, 57 °C for 0:20 min, 72 °C for 1:30 min, and a final extension at 72 °C for 5 min. The resulting products were separated on 1.5% agarose gels using a Tris/acetate/EDTA buffer system and visualized with SYBR Safe (ThermoFisher Scientific). A subset of the reactions was sub-cloned into pCR2.1-TOPO TA (ThermoFisher Scientific) and sequence-validated (Retrogen Inc., San Diego, CA, USA). Consensus sequences for the four ASCTRs have been deposited with GenBank under accession numbers MW653946-MW653949. Gel images were obtained with an Azure 200 gel imaging workstation (Azure Biosystems, Dublin, CA) and then processed in Adobe Photoshop v21.2.6 (Adobe, San Jose, CA, USA).

### 4.4. Construction of Expression Plasmids

To examine the cellular localization of the predicted Homam-ASTCRs, fluorescent chimeras of the respective receptors tagged at their C terminal ends with enhanced green fluorescent protein (EGFP) were generated via overlap extension PCR [76] and then cloned into the pIB/v5-His insect cell expression vector (ThermoFisher Scientific). Overlap extension PCR was performed using KOD-Hot Start DNA polymerase (EMD Millipore, San Diego, CA, USA) with sequence-validated plasmids and both target specific and chimeric primers (Table S7). The control EGFP expression vector was generated previously [77]. Initial thermocycler conditions consisted of 95 °C for 2 min followed by 25 cycles at 95 °C for 0:20 min, 58 °C for 0:20 min, 70 °C for 0:30 min, and a final extension at 72 °C for 5 min. Final thermocycler conditions consisted of 95 °C for 2 min followed by 27 cycles at 95 °C for 0:20 min, 56 °C for 0:20 min, 70 °C for 1:30 min, and a final extension at 72 °C for 5 min. Non-fluorescent expression vectors encoding Homam-ASTCR1, 2, and 4 were similarly generated. Clones were sequence verified as before.

### 4.5. Transient Expression and Binding Potential of ASTCR-EGFP Chimeras 

For transient expression analyses, cultured *Trichoplusia ni* (Tni) insect cells (Allele Biotech Inc., San Diego, CA, USA), an ovarian-derived cell line [44], were transfected with expression plasmids (see above) as previously described [78]. Briefly, cells maintained as a monolayer at 28 °C in Ex-cell 420 serum-free media (Millipore Sigma, St. Louis, MO, USA) were seeded onto 35 mm #1.5 glass bottom dishes (Matsunami Glass USA Inc., Bellingham, WA, USA) and then transfected with 2 μg sequence-verified plasmid using 8 μL cellfectin II (ThermoFisher Scientific). After 5 h, the transfection media was replaced, and the cells were maintained at 28 °C for 48 h. 

To assess potential ligand binding, the cells transiently expressing the ASTCR-EGFP chimeras were examined for their ability to bind a synthetic analog of Homam-AST-C I tagged at its N terminus with the red fluorescent dye, carboxytetramethylrhodamine (TAMRA; Em max ∼580 nm). The cells were pre-chilled for 5 min at 4 °C and then incubated in the dark for 1 h at 4 °C in IPL-41 insect medium (Thermo Fisher Scientific) with 500 nM TAMRA-AST-C I alone or in combination with unlabeled peptides: AST-C I (5 μM), AST-C III (5 μM), or a peptide fragment corresponding to amino acids 21–36 of the *Drosophila melanogaster* sex peptide (5 μM). Cells were washed with ice-cold IPL-41 and then placed in 2 mL IPL-41 media supplemented with sucrose (0.45 M final concentration). The hypertonicity of the media inhibits clathrin-mediated receptor internalization [79]. Cells were imaged using a 60× phase contrast water immersion objective/NA 1.2 on a FluoView FV10i laser scanning confocal microscope (Olympus Scientific Solutions, Waltham, MA., USA) equipped with filters for EGFP (Em = 489 nm/Ex = 510 nm) and TAMRA fluorescence (TRITC filter; Em = 546 nm/Ex = 579 nm). The images were subsequently processed using ImageJ (Schneider et al. 2012) and Adobe Photoshop v21.2.6. The synthetic TAMRA-AST-C I (5-TAMRA-QIRYHQCYFNPISCF; disulfide between the two Cys) and *D. melanogaster* sex peptide fragment (DKWCRLNLGPAWGGRC; disulfide between the two Cys) were custom synthesized and purified to > 95% purity (United Biosystems Inc., Herndon, VA, USA). The unlabeled AST-C I (QIRYHQCYFNPISCF; disulfide between the two Cys) and III (GNGDGRLYWRCYFNAVSCF; disulfide between the two Cys) were likewise custom synthesized (GenScript Biotech., Piscataway, NJ, USA).

### 4.6. Stable Expression of Non-Tagged Homam-ASTCRs in Cultured Insect Cell Lines

For stable expression analyses, cultured Sf9 insect cells (Gibco., San Diego, CA, USA), an ovarian-derived cell line from fall armyworm (*S. frugiperda*), were transfected with pIB/V5-His expression plasmids for untagged Homam-ASTCR1, Homam-ASTCR2, and Homam-ASTCR4. Sf9 cells maintained as a monolayer at 28 °C in Graces insect medium (Gibco/ThermoFisher Scientific, Grand Island, NY, USA) supplemented with 10% fetal bovine serum (Gibco/ThermoFisher Scientific) were seeded onto 35 mm tissue culture dishes (CELLTREAT Scientific Products, Pepperell, MA, USA) and then transfected with sequence-verified plasmids as before. After 5 h, the transfection medium was replaced, and the cells were maintained at 28 °C for 48 h. Cells were selected with 250 μg/mL blasticidin S hydrochloride (ThermoFisher Scientific) for 1 week and then maintained in Graces media/10% fetal bovine serum supplemented with 25 μg/mL blasticidin S hydrochloride. To confirm transgene expression, 1.5 mL aliquots of each polyclonal line along with non-transfected Sf9 cells were pelleted and total RNAs were isolated and purified using TRI Reagent (ThermoFisher Scientific) with RNeasy mini kit spin columns (Qiagen, Germantown, MD, USA) on a QIAcube automated nucleic acid isolation system (Qiagen). cDNAs were generated from 500-ng DNase I (New England Biolabs, Ipswich, MA, USA)-treated RNAs using a SuperScript III First-Strand Synthesis System as before. PCR was performed using Sapphire Amp Fast PCR Master Mix and primers (Table S7) designed to amplify ~ 500-bp fragments of each ASTCR (ASTCR1—nt 90–623; ASTCR2—nt 20–568; ASTCR4—nt 446–979) and a 514-bp fragment (nt 258–771) of the *S. frugiperda* actin transcript (HQ008727). Thermocycler conditions consisted of 95 °C for 2 min followed by 35 cycles at 95 °C for 0:20 min, 56 °C for 0:20 min, 72 °C for 0:30 min, and a final extension at 72 °C for 5 min.

To assess ligand binding, the polyclonal Sf9 lines stably expressing Homam-ASTCR1, Homam-ASTCR2, and Homam-ASTCR4 were examined for TAMRA-AST-C I binding as above but with 2.5 μM TAMRA-AST-C I. Cells were imaged as before on a FluoView FV10i laser scanning confocal microscope and processed with ImageJ. To assess receptor activation, intracellular cAMP levels were determined using a cyclic AMP select ELISA kit (Cayman Chemical, Ann Arbor, MI, USA). Polyclonal ASTCR and non-transfected Sf9 cells were incubated for 30 min at room temperature in IPL-41 media with 200 μM 3-isobutyl-1-methylxanthine (IBMX; Cayman Chemical, Ann Arbor, MI, USA) and 10 μM forskolin (Cayman Chemical) alone or in combination with 2 μM synthetic Homam-AST-C I or III. Cells were then lysed in 0.1 N HCl for 20 min and pelleted. cAMP levels in the supernatant were determined by measuring Abs at 425 nm on a Synergy H4 Hybrid Multi-Mode Microplate Reader (Biotek Instruments, Winooski, VT, USA). To facilitate comparisons across experiments, data were assessed as a percentage of maximal forskolin stimulation. Statistical analyses were performed in GraphPad Prism v.8 (GraphPad Software, San Diego, CA, USA) using ANOVA with an uncorrected Fisher’s LSD test, *p* < 0.05.

### 4.7. Physiological Recording and Analysis

The lobsters were anesthetized on ice for 30–60 minutes; the lobster heart was then removed from the body and pinned to a Sylgard 184-coated dish (Dow Corning, Midland, MI, USA) filled with cold lobster physiological saline (composition in mM: 479.12 NaCl, 12.74 KCl, 13.67 CaCl2, 20.00 MgSO4, 3.91 Na2SO4, 11.45 Trizma base, and 4.82 maleic acid; pH 7.45). The posterior artery was cannulated, so that saline could be perfused through the heart. The saline was maintained at 10–12 °C using a Peltier temperature regulator (CL100 bipolar temperature controller and SC-20 solution heater/cooler; Warner Instruments, Hampden, CT, USA). The perfusion rate through the heart was held constant at 2.5 mL/min by a Rabbit peristaltic pump (Gilson, Middleton, WI, USA); a second channel was directed across the top of the heart to maintain temperature. The anterior arteries were tied to a FT03 force transducer (Grass Natus Technologies, Pleasanton, CA, USA), the output of which was amplified using an ETH-250 Bridge amplifier (CB Sciences, Dover, NH, USA) with a high pass filter set to 4 Hz, and a Brownlee 410 instrumentation amplifier (Brownlee Precision, San Jose, CA, USA). Contractions of the heartbeat were recorded at a sampling frequency of 10,000 Hz on a P.C. computer using a 1401 data acquisition interface and CED Spike2 software v7 (Cambridge Electronic Design Limited, Cambridge, England, UK). After a 1 h period of stabilization, each AST-C isoform was perfused through the system with 50 min washes of saline between isoforms. Isoforms were introduced in pseudorandom order across preparations. The physiological responses were analyzed by calculating the amplitude and frequency of the heartbeat over the course of the recording in Spike2 using functions built into the program. The percentage changes in amplitude and frequency were then calculated by determining the average amplitude and frequency of a set of 50 heartbeats at three timepoints: baseline before the peptide was perfused, during the peak response of the heart to the peptide, and after the peptide had been entirely washed out and the heartbeat had returned to baseline amplitude and frequency. Only hearts that returned to approximately baseline activity after perfusion with each peptide were used in the analysis.

### 4.8. CG RNA Sequencing and Analysis

After physiological recordings, CGs were dissected out of the heart and RNA was extracted using Direct-zol RNA Micro Prep kits (Zymo Research, Irvine, CA, USA). The quality and quantity of the RNA was confirmed using an Agilent 2100 Bioanalyzer (Agilent Technologies, Santa Clara, CA, USA). Paired-end Illumina RNA sequencing was performed by Georgia Genomics and Bioinformatics Core at the University of Georgia (Athens, GA) on the RNA from the CGs of 24 different lobsters for which the physiological responses to AST-C I and III had been recorded. 

Quality control checks were performed on the sequencing data using FastQC [80] (version 0.11.9, Barbraham Bioinformatics, U.K.). The data were cleaned using Trimmomatic (version 0.39) [81]. Trimmomatic parameters removed any bases at the beginning or end of the reads if their quality score was below three, set a target length of 40 bases using the “Max.info” function, and eliminated any reads that had an average Phred Score below 28 or were shorter than 25 bases. This process removed 20–25% of the reads due to low quality. After the data were cleaned, the quality of the remaining reads was confirmed with FastQC.

The RNA-seq data from the CGs of the 24 different lobsters were then mapped to a master *H. americanus* transcriptome for reference. The need for a master transcriptome was determined when we observed that the transcript for ASTCR1 was not present in the CG-specific transcriptome [33]. Since ASTCR1 was successfully cloned from CG RNA, we suspected that this transcriptome was incomplete. Therefore, we created a master transcriptome by concatenating four pre-existing *H. americanus* transcriptomes to ensure better coverage. The four transcriptomes incorporated in the master transcriptome are a CG transcriptome (Accession Number: GGPK00000000; BioProject Number: PRJNA412549), a brain transcriptome (Accession Number: GFUC00000000; BioProject Number: PRJNA379629), an eyestalk transcriptome (Accession Number: GFDA00000000; BioProject Number: PRJNA338672), and a mixed neural tissue transcriptome (Accession Number: GEBG00000000; BioProject Number: PRJNA300643) [33,35,37,82]. Before concatenating the four transcriptomes, redundancies were eliminated using CD- HIT-EST [83]. CD-HIT-EST clustered 67,690 transcripts from the mixed neural tissue transcriptome into 67,334 clusters, 146,106 transcripts from the eyestalk transcriptome into 102,718 clusters, 150,579 transcripts from the brain transcriptome into 106,998 clusters, and 189,952 transcripts from the cardiac ganglion transcriptome into 127,191 clusters. After concatenation, CD-HIT-EST was run on the new master transcriptome in order to remove any further redundancies that arose from combining these transcriptomes.

Kallisto [84] pseudoalignment of the CG reads to the transcriptome was performed. This generated estimated count and transcripts per million (tpm) data on the expression of each of the transcripts from the transcriptome in the cardiac ganglion RNA samples.

### 4.9. Spearman Correlations of AST-C Receptors

Spearman correlations were performed in R (version 1.2.1335, RStudio, Inc, Boston, MA, USA) to analyze the relationship between the expression of ASTCR1-4 and the physiological response to AST-C I. In addition to analyzing the expression of individual receptors, the ratio of expression of each receptor to every other receptor was calculated for each sample. Spearman correlations were performed to analyze the relationship between these ratios and the physiological responses.

## Data Availability

The data presented in this study are available in this article, including supplementary figures and tables, or at NCBI: cardiac ganglia accession GGPK00000000 in BioProject PRJNA412549; brain accession GFUC00000000 in BioProject PRJNA379629; eyestalk accession GFDA00000000 in BioProject PRJNA338672; and mixed neural tissue accession GEBG00000000 in BioProject PRJNA300643.

## Supplementary Materials

The following are available online at https://www.mdpi.com/article/10.3390/ijms22168703/s1; Figure S1: Phylogenetic analysis of H. americanus ASTCRs with ASTCR-like receptors from diverse arthropods; Figure S2: Neighbor joining phylogenetic tree of H. americanus ASTCRs with the full complement of annotated peptide receptors from Drosophila melanogaster and Cancer borealis, Figure S3: Localization and binding potential of heterologously expressed Procambarus clarkii ASTCR4; Figure S4: H. americanus ASTCR transgene expression in stably transformed polyclonal Sf9 insect cells; Figure S5: The ratio of expression of ASTCR1 to ASTCR4 in the cardiac ganglion shows a nonsignificant correlation with physiological response to AST-C I; Table S1: Bioinformatic analysis of Homarus americanus ASTCRs; Table S2: Top 5 BLASTx hits using putative Homarus americanus ASTCRs as query sequences; Table S3: BLASTn analysis of Homarus americanus ASTCR sequences against a cardiac ganglia specific transcriptomic dataset; Table S4: Spearman correlation statistics for differential receptor expression; Table S5: tBLASTn analysis of tissue-specific Homarus americanus transcriptomic datasets; Table S6: Accession numbers of proteins used in maximum likelihood phylogenetic analyses; Table S7: Oligonucleotide primers used in this study; Table S8: Accession numbers of proteins used in phylogenetic analyses using the full receptor complement from two arthropods.
