# Supplementary figures and images for: Does Differential Receptor Distribution Underlie Variable Responses to a Neuropeptide in the Lobster Cardiac System?
Source: Int J Mol Sci. 2021 Aug 13;22(16):8703. doi: 10.3390/ijms22168703 (PMC8395929; doi:10.3390/ijms22168703)

A)

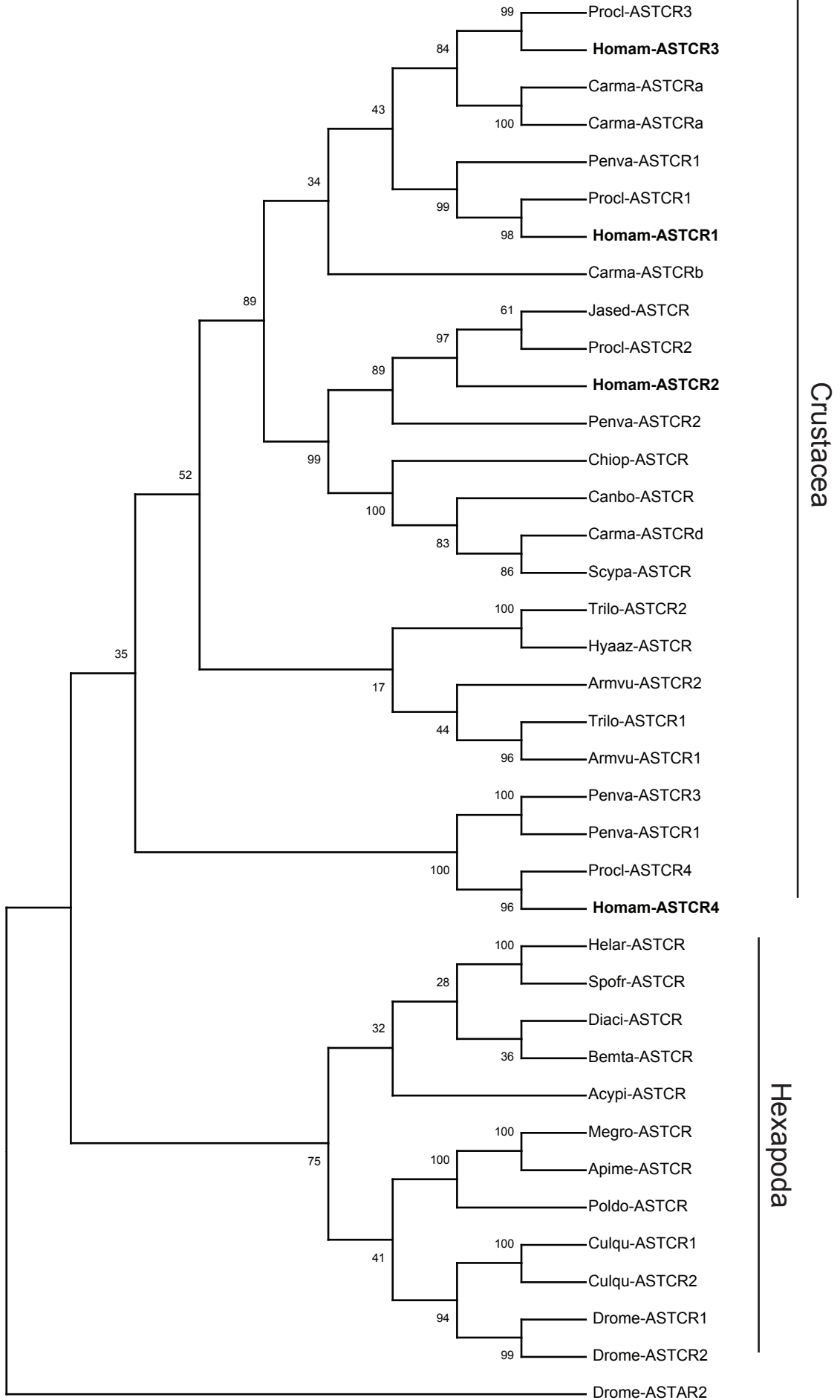

B)

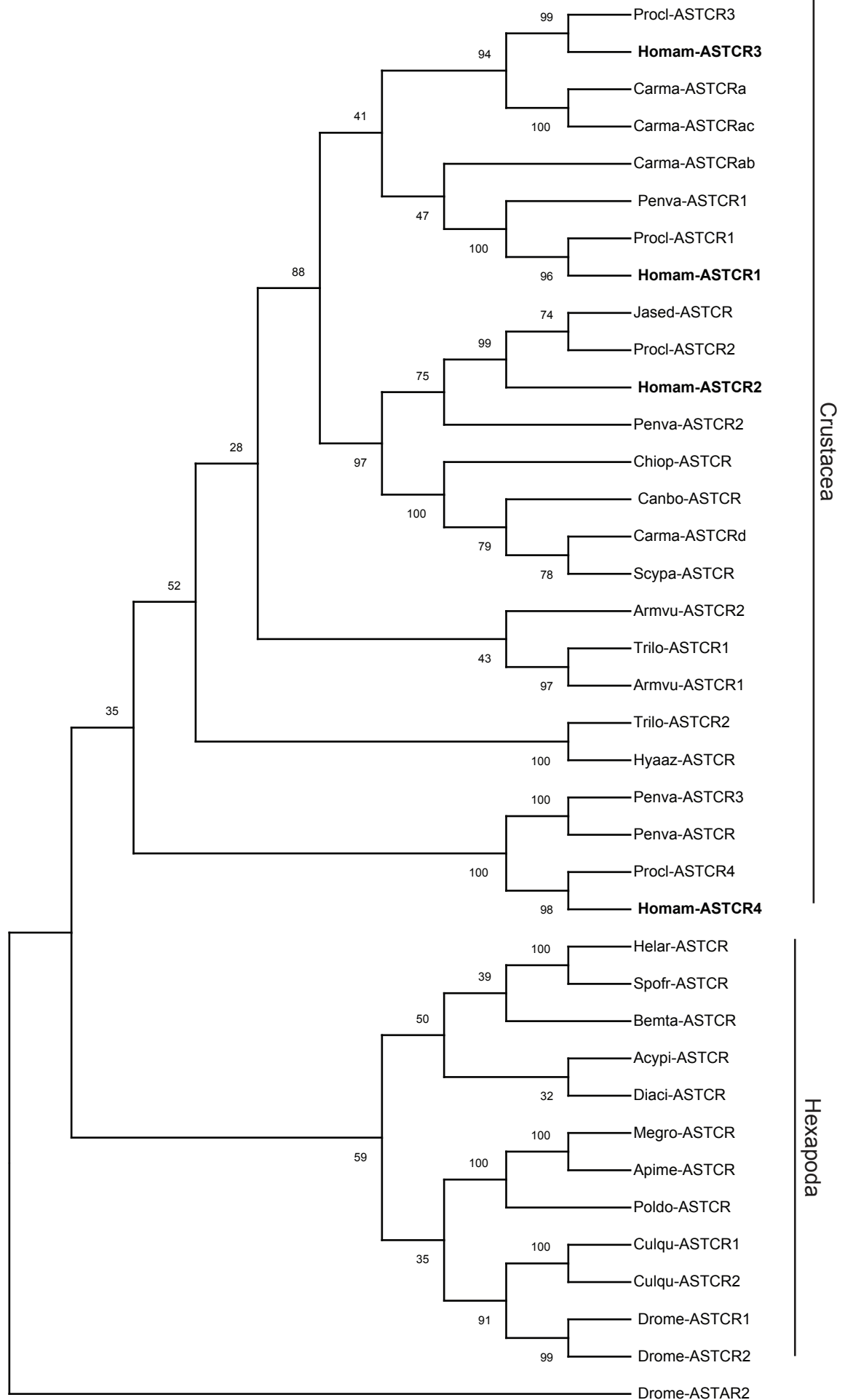

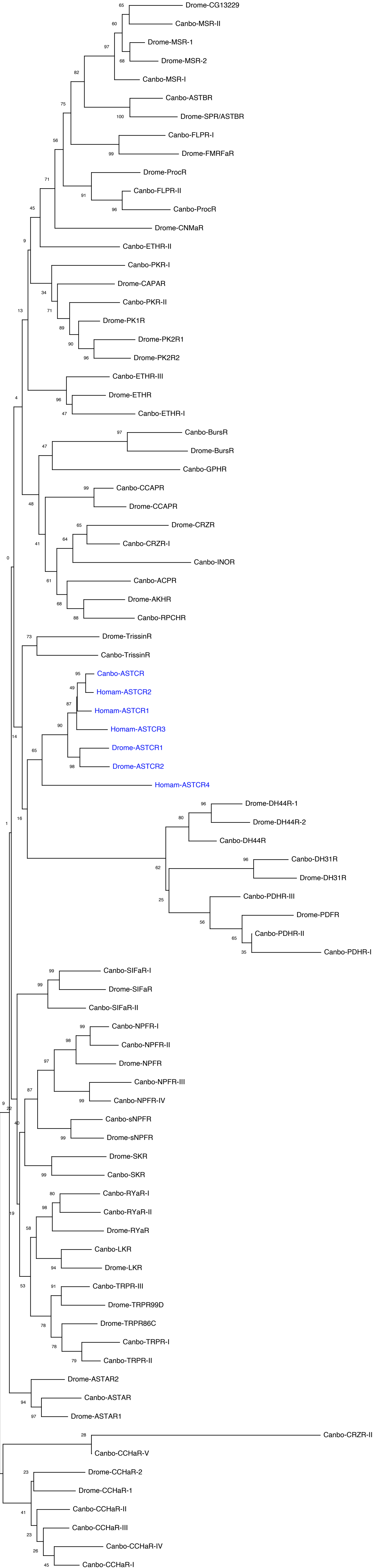

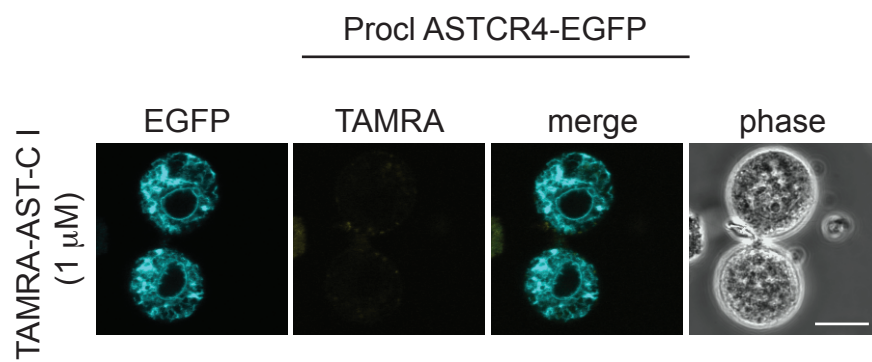

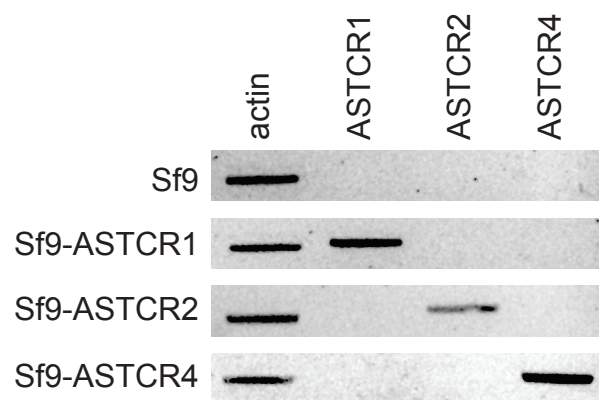

ASTCR1 : ASTCR4 Expression (tpm)

$R = -0.37$  ,  $p = 0.075$

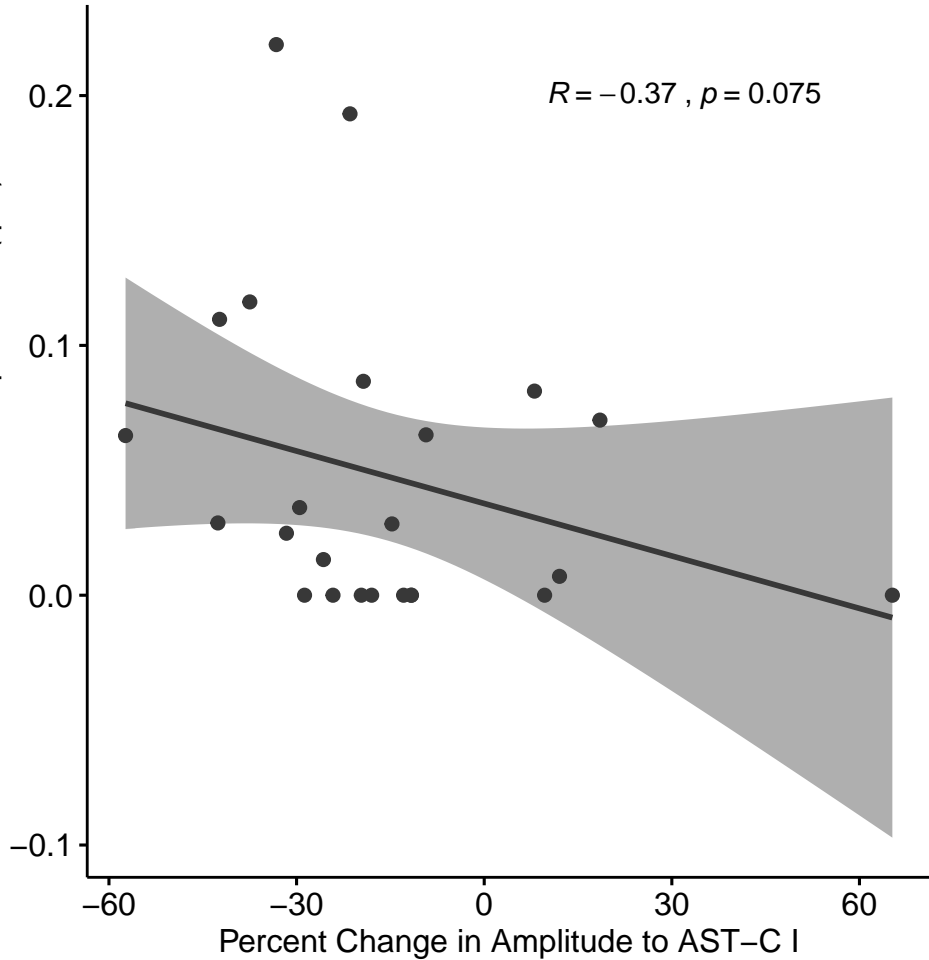

Supplement: Supplementary file 1 [file ijms-22-08703-s001.zip › FigS1-5.pdf]
